# Collagen Scaffolds in Bone Sialoprotein-Mediated Bone Regeneration

**DOI:** 10.1155/2013/812718

**Published:** 2013-03-31

**Authors:** Thomas E. Kruger, Andrew H. Miller, Jinxi Wang

**Affiliations:** ^1^Harrington Laboratory for Molecular Orthopedics, Department of Orthopedic Surgery, University of Kansas Medical Center, Kansas City, KS 66160, USA; ^2^Department of Biochemistry and Molecular Biology, University of Kansas Medical Center, Kansas City, KS 66160, USA

## Abstract

Decades of research in bioengineering have resulted in the development of many types of 3-dimentional (3D) scaffolds for use as drug delivery systems (DDS) and for tissue regeneration. Scaffolds may be comprised of different natural fibers and synthetic polymers as well as ceramics in order to exert the most beneficial attributes including biocompatibility, biodegradability, structural integrity, cell infiltration and attachment, and neovascularization. Type I collagen scaffolds meet most of these criteria. In addition, type I collagen binds integrins through RGD and non-RGD sites which facilitates cell migration, attachment, and proliferation. Type I collagen scaffolds can be used for bone tissue repair when they are coated with osteogenic proteins such as bone morphogenic protein (BMP) and bone sialoprotein (BSP). BSP, a small integrin-binding ligand N-linked glycoprotein (SIBLING), has osteogenic properties and plays an essential role in bone formation. BSP also mediates mineral deposition, binds type I collagen with high affinity, and binds **α**v**β**_3_ and **α**v**β**_5_ integrins which mediate cell signaling. This paper reviews the emerging evidence demonstrating the efficacy of BSP-collagen scaffolds in bone regeneration.

## 1. Introduction

Collagens are a group of closely related proteins that comprise the most abundant proteins found in mammals representing 25–35% of the total body protein [1]. To date, at least 28 different types of collagen encoded by 45 genes have been identified [2, 3]. Collagen is found in cartilage, bone, intervertebral discs, blood vessels, tendons, ligaments, skin, and cornea and is the main component of extracellular matrix. Various types of collagens are synthesized by fibroblasts, smooth muscle cells, chondrocytes, osteoblasts, endothelial cells, epithelial cells, myoblasts, neural retinal cells, and notochord cells. Type I collagen is the most abundant of all 28 known collagens and is the most abundant type found in bone constituting >90% of the organic mass of bone [4]. 

 Collagens have many unique properties that make them particularly useful as scaffolds for facilitating tissue regeneration and/or site-specific drug delivery. For example, collagen has low antigenicity, low toxicity, a high affinity for water and is biodegradable [5, 6]. Also, collagen contains RGD (Arg-Gly-Asp) and non-RGD domains which bind cell surface-associated integrins [7, 8] thus facilitating cell migration [9], attachment [10–12], proliferation [13–15], and differentiation [12, 16]. As such, collagen-based materials have been used for sutures, implants, wound dressings, and DDS and are being developed as matrices/scaffolds with or without bioactive proteins/peptides and cells that potentiate regeneration of both soft and hard tissues such as skin or bone [6, 17].

## 2. Collagen Structure

Type I collagen consists of two identical *α*1 chains and one different *α*2 chain, hence the chain designation for type I collagen is denoted [*α*1(I)]_2_ *α*2(I)]. A single peptide chain of collagen (*α*-chain) is comprised of 33% glycine (G), 10% proline (P), and 10% hydroxyproline (HyP) in a distinctive repeating pattern of (glycine-X-Y)_*n*_, where X is frequently proline and Y is frequently 4-hydroxyproline (some 3-hydroxyproline or 5-hydroxylysine). This unique amino acid sequence confers a “left-handed” polyproline II type (PPII) helical structure to each *α*-chain [18]. During genesis of collagen fibers, three parallel “left-handed” PPII helical *α*-chains “self-assemble” around each other with a one amino acid stagger in a “right-handed” fashion to form a right-handed triple helix referred to as a *tropocollagen* molecule [18–23]. The staggered arrangement of the 3 peptide backbones of tropocollagen aligns the G residues amino groups on one peptide chain with the P residues' carbonyl oxygens on one of the other peptide chains allowing the formation of hydrogen bonds between all three peptide chains down the entire length of the chains. The resulting hydrogen bonds formed in this way between all three peptide chains help maintain the stability of tropocollagen. After triple helix formation, but before fibrillogenesis can occur, propeptides from both termini are removed by specific peptidases from the tropocollagen molecule leaving a triple helix flanked by short nonhelical *telopeptides* (the most antigenic portion of collagen). 

**Figure 1:**
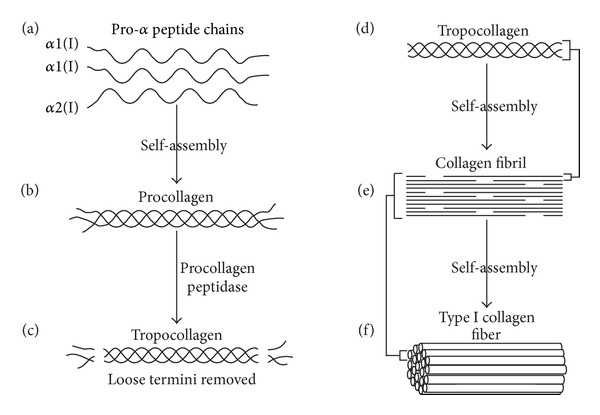
The process of type I collagen synthesis. (a) Two identical *α*1(I) and one *α*2(I) peptide chains self-assemble to form procollagen (b). (c) Procollagen peptidase removes loose termini to create a type I tropocollagen molecule (d). Tropocollagen molecules self-assemble to form a growing collagen fibril (e). Self-assembly of collagen fibrils forms a type I collagen fiber (f).

 A collagen fiber segment or fibril is comprised of five parallel tropocollagen molecules which have staggered ends thus facilitating and strengthening a “growing” collagen fiber made up of multiple “head to tail” tandem collagen fiber segments (Figure 1). As tropocollagen molecules associate to form fiber segments, *inter*tropocollagen hydrogen bonds form between terminal HyP carbonyl oxygens in a peptide of one tropocollagen and the terminal hydroxyl hydrogen of a HyP in a peptide of another tropocollagen molecule. Also at the ends of the tropocollagen segments, hydrogen bonds form between the hydrogen of HyP in one peptide and a G carbonyl oxygen of the other peptide [18]. The left-handed helical direction of collagen's polypeptide chains combined with the right-handed helix of tropocollagen converts a *longitudinal tensional* force to a *lateral compressional* force on the tropocollagen triple helix. Additionally, the triple helical structure of collagen protects it from enzymatic degradation, facilitates cell adhesion, and plays a key role in assembly of the extracellular matrix (for review of collagen structure see [18]). 

 The physical and chemical properties of collagen have elicited efforts to develop collagen-based DDS and biomedical matrices for neotissue generation. Mafi and colleagues define the ideal collagen-based scaffold as one which is nontoxic, nonantigenic, three-dimensional, biocompatible, biodegradable, highly porous (allowing cell and nutrient influx and waste material efflux), conducive for cell attachment, proliferation and differentiation, osteoconductive, and mechanically flexible and elastic [24].

### 2.1. Collagen-Based Drug Delivery Systems

The use of collagen-based materials as DDS spans nearly 45 years and includes delivery of antibiotics, steroids, anticoagulants, antineoplastics, immunosuppressants, growth factors, cytokines and gene therapeutics (e.g., plasmid DNA) (for review see [25]). The goals of these efforts have been to (1) deliver a bioactive substance *directly* to the appropriate site (without oral or systemic delivery), (2) achieve an *effective concentration* at the appropriate *time* and *duration*, (3) maintain suitable biodegradability negating additional surgical intervention, and (4) prevent infection, promote wound healing and tissue regeneration, or deliver chemotherapeutics site-specifically. In attempts to control these parameters, collagen matrices in the form of films, sheets, wafers, discs, gels, sponges, 3D scaffolds, and nanofibers (alone and in combination with a plethora of natural and synthetic fibers as well as ceramics) have been studied in a number of *in vitro* and *in vivo* applications (for review see [25]). Recently, Gils et al. developed a pH sensitive “intelligent” hydrogel DDS comprised of hydrolyzed collagen combined with polymers of acrylamide and itaconic acid for oral delivery of the angiotensin II receptor antagonist Valsartan [26]. Also, Kojima et al. demonstrated suppressed tumor growth and metastatic activity of MDA-MB-231 breast cancer cells *in vivo* using a pH sensitive DDS comprised of a dendrimer/collagen gel hybrid conjugated with doxorubicin [27]. Collagen-based materials are not only being used as viable forms of DDS but are also being developed as tissue engineering scaffolds. 

### 2.2. Tissue Engineering

The biocompatible properties of collagen have resulted in the use of collagen as a matrix or scaffold for tissue regeneration. Native collagen and denatured collagen (gelatin), alone or in combination with other natural and synthetic polymeric fibers as well as ceramics, have been assessed for their inherent scaffold characteristics. These “collagen hybrids” are in part designed to control release/delivery of bioactive substances, prolong the biodegradation of the scaffold, or overcome collagen's lack of mechanical strength in certain hard tissue (e.g., skeletal tissue) applications. While different types of extracellular matrix proteins such as other collagens, elastin, hyaluronan, and glycosaminoglycans (GAG) have been used for scaffolds, type I collagen is the most prevalent scaffold material due to its biocompatibility and availability [28]. 

 Investigators studying the biomedical application(s) of collagen-based matrices for tissue engineering have utilized both cell-free systems and matrices seeded *in vitro* with specific cell types. Cell-free systems usually encompass immobilization of proteins or other bioactive substances (e.g., growth factors) directly within the collagen-based matrix in attempts to stimulate histogenesis, recruit regenerative cells into the tissue, or block unwanted cell influx. For example, acellular type 1 collagen-heparin scaffolds containing fibroblast growth factor 2 (FGF2) and vascular endothelial growth factor (VEGF) can stimulate angiogenesis, neovascularization and vascular tissue regeneration *in vivo* [29, 30]. To reduce wound contraction and scarring following cleft palate surgery, Jansen et al. developed interferon-*γ*-loaded collagen scaffolds that significantly diminished myofibroblast influx/differentiation following palate surgery in Wistar rats [31]. 

 An alternative approach for tissue regeneration involves culturing specific cell types directly on the collagen-based matrix prior to *in vivo* application. Many different cell types have been cultured on collagen-based scaffolds and subsequently assessed both *in vitro* and *in vivo* for the functional capacity to regenerate specific tissues. To date, numerous cell types including mesenchymal stem cells, fibroblasts, keratinocytes, chondrocytes, osteoblasts, and more have been seeded onto collagen scaffolds for regenerative applications in a variety of tissues including skin, cornea, cardiovascular, urogenital, neural, and osteochondral tissues (for detailed review see [28, 32, 33]). The use of 3-D scaffolds for specific cell culture may provide certain advantages over cell cultures grown in monolayers. For example, chondrocytes cultured on porous 3-D collagen sponges exhibit sustained gene expression (aggrecan core protein and type II collagen) and produce greater amounts of extracellular matrix proteins as compared to chondrocytes grown in monolayers [28, 34, 35]. However, variations in culture conditions and matrix composition may result in different experimental results. For example, while type I collagen promotes proliferation and osteoblastogenesis of human mesenchymal stem cells *in vitro *[36], mesenchymal stem cells cultured on type I collagen-GAG scaffolds prior to implantation into rat calvarial defects provide no additional benefit and may actually be deleterious when compared to the reparative effects of collagen-GAG scaffolds alone or particulate autogenous bone [37]. Thus the particular combination of collagen-based scaffold and cell type used in scaffold seeding may not be inherently predictable for a given application but in fact requires careful experimental assessment prior to development for use *in vivo*.

### 2.3. BSP-Collagen in Osteoblast Differentiation and Bone Regeneration

Human bone sialoprotein (BSP) is a 33 kDa (apparent molecular weight of 60–70 kDa due to extensive posttranslational modifications) noncollagenous glycoprotein in mineralized tissues such as bone, dentin, cementum, and calcified cartilage [38]. During bone morphogenesis, BSP is produced by osteoblasts, osteoclasts, osteocytes and hypertrophic chondrocytes. Through unique-binding domains, BSP may be involved in cell attachment and signaling, hydroxyapatite (HA) nucleation, and binding of type I collagen [39–42]. Tye et al. has identified the type I collagen-binding domain of recombinant rat BSP as an N-terminal stretch of amino acids (aa) corresponding to aa 19–46, a region which is highly conserved between rat, mouse, pig, cow, and human BSP where 20 aa out of 27 are identical [42]. Based on these studies, the binding of BSP to type I collagen involves predominantly hydrophobic interactions and to a lesser degree electrostatic, since increasing ionic strength or lowering the pH below 7.0 only partially abrogates BSP-collagen binding. The studies of Baht et al. extend these observations and demonstrate higher BSP-binding affinities for *α*-helical domains of collagen such as those found in triple helical type I collagen (Kd ~ 1.3 × 10^−8^), atelo type I collagen (Kd ~ 1.3 × 10^−8^), and fibrillar type I collagen (Kd ~ 1.2 × 10^−8^) as compared to denatured type I collagen (gelatin; Kd ~ 4.5 × 10^−8^) devoid of *α*-helical structure [43]. 

 Experimental evidence demonstrates that BSP plays an essential role in differentiation of osteoblasts from bone marrow cells (BMCs) cultured on type I collagen *in vitro* [44]. For example, bone marrow cells cultured for 3 weeks on type 1 collagen express osteoblast differentiation markers/phenotypes such as increased alkaline phosphatase (ALP) activity, enhanced osteocalcin synthesis, elevated intracellular cAMP in response to parathyroid hormone (PTH), and BSP production/secretion (a marker of osteoblast differentiation) as opposed to bone marrow cells cultured on plastic [44]. Also in these studies, HA mineralization began in bone marrow-type I collagen cultures within 2 weeks as evidenced by the formation of calcified nodules. The expression of osteoblastic markers was abrogated by coculture with a monoclonal anti-BSP antibody [44].

Recently, investigators have examined the *in vitro* and *in vivo* osteogenic properties of a BSP-derived collagen-binding (CB) peptide corresponding to the aa sequence 35–62 of rat BSP [45]. In these studies, CB peptide specifically bound type I collagen and stimulated human osteosarcoma (HOS) cell differentiation into osteoblasts as determined by upregulation of ALP, type I collagen, and osteopontin gene expression after 14 days coculture with 20, 40, and 80 *μ*g/mL CB peptide *in vitro*. In addition, CB peptide (40 *μ*g/mL) treatment of HOS cells stimulated the activation of mitogen activated protein kinase (MAPK) and protein kinase B (Akt) pathways *in vitro*, which are known to be activated during cell differentiation [46–48]. *In vivo* studies demonstrate that CB-HA implants containing 6 mg CB peptide placed into surgically created 8 mm rabbit calvarial defects significantly stimulates more new bone growth within 2 weeks after-surgery as compared to untreated or HA scaffolds alone [45]. A study using primary bone-derived cells on quartz surfaces grafted with a peptide containing a RGD-sequence unique to BSP demonstrated that this peptide significantly enhanced the strength of bone cell adhesion [49]. 

**Figure 2:**
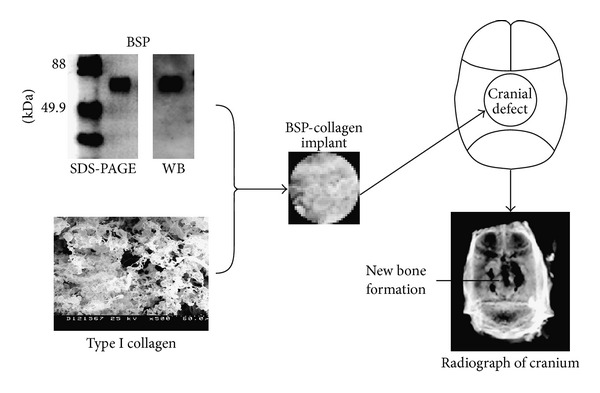
BSP-collagen implant preparation and implantation. A diagram shows the process of BSP-collagen implant preparation, BSP-collagen implantation into a rat calvarial bone defect and new bone formation in the defect at day 30 after implantation of BSP-collagen.

 Our* in vivo* studies demonstrate that BSP-collagen implants placed into surgically created 8 mm rat calvarial defects stimulate osteoblast differentiation and bone repair [50, 51]. BSP-collagen (but not collagen alone) upregulated the expression of genes associated with early osteoblast differentiation, as early as 4 days after implantation. In this model, cell proliferation, matrix mineralization, and vascular invasion extended into the central regions of the BSP-collagen implants (but not collagen alone implants) by day 7 after surgery. By day 10 after implantation, osteoblast differentiation was more active in BSP-collagen implants when compared to BSP-gelatin or collagen alone as determined by increased alkaline phosphatase (ALP) and osteocalcin (OCN) expression. By days 21–30, defects receiving BSP-collagen implants demonstrated significant new bone formation and remodeling in both the central regions of the calvarial defect and the areas adjacent to the host bone, whereas defects receiving collagen alone displayed new bone only in the areas near the host bone (Figure 2). Taken together, the data suggests BSP-collagen implants are an ideal candidate for tissue engineering of bone defects since BSP (1) plays an essential role in osteoblast differentiation, (2) binds type I collagen with high affinity via an N-terminal domain, (3) binds *α*v*β*_3_ and *α*v*β*_5_ integrins which mediates cell signaling and differentiation [52], (4) combined with *collagen* which facilitates cell migration, attachment, proliferation and differentiation through RGD and non-RGD binding of integrins. 

## 3. Conclusions and Future Perspectives

Studies in the field of tissue engineering have resulted in numerous synthetic and natural materials that are useful in regeneration of both soft and hard tissues. After years of matrix development and evaluation, a number of criteria have emerged which define the “ideal” DDS or tissue engineering scaffold. Certainly, 3-D highly porous scaffolds that are biocompatible and biodegradable promote cell influx and proliferation, stimulate neovascularization into the graft, are capable of preseeding with progenitor cells, or are supplemented with bioactive substances are all highly desirable scaffold attributes. While no single scaffold material will achieve all the desired properties, the availability, low antigenicity, and overall biocompatibility of type I collagen make it a useful matrix for many applications, including cartilage and bone regeneration. The central role that BSP plays in bone formation through its distinct multifunctional multiple-binding domains involved in cell attachment and signaling, HA binding and nucleation, and specific binding of type 1 collagen make BSP-collagen scaffolds an excellent choice for bone regeneration. An apparent limitation of collagen is lack of mechanical strength which can be overcome by “blending” the matrix with materials that confer increased strength to the scaffold making it more suitable for load bearing applications. Supplementation of BSP-collagen with HA, secreted protein acidic and rich in cysteine (SPARC), or intrafibrillar silicification could facilitate matrix mineralization and enhance the mechanical strength of collagen scaffolds [53–55], which may make a BSP-collagen matrix more suitable for load-bearing applications.
